# A feasibility study of deep learning-based segmentation of the inferior alveolar nerve on magnetic resonance neurography

**DOI:** 10.1038/s41598-026-45392-6

**Published:** 2026-04-01

**Authors:** Yoon Joo Choi, Sujeong Han, Chena Lee, Kug Jin Jeon, Haesung Oh, Sang-Sun Han, Jaesung Lee

**Affiliations:** 1 Department of Oral and Maxillofacial Radiology, Yonsei University College of Dentistry, 50-1 Yonsei-ro Seodaemun-gu, Seoul, 03722, Republic of Korea; 2 Department of Artificial Intelligence, Chung-Ang University, Seoul, Republic of Korea; 3 Institute for Innovation in Digital Healthcare, Yonsei University Health System, Seoul, Republic of Korea; 4 AI/ML Innovation Research Center, Chung-Ang University, Seoul, Republic of Korea

**Keywords:** Artificial intelligence, Deep learning, Magnetic resonance neurography, Image segmentation, Inferior alveolar nerve, Computational biology and bioinformatics, Diseases, Engineering, Health care, Mathematics and computing, Medical research

## Abstract

The inferior alveolar nerve (IAN) is the major sensory nerve innervating the mandibular region, and its automatic segmentation is crucial. It has been indirectly identified on the mandibular canal, which surrounds the IAN, using computed tomography (CT) and cone-beam computed tomography (CBCT). Magnetic resonance neurography (MRN) is an imaging technique designed for nerve visualization, facilitating discrimination of small peripheral nerves from surrounding soft tissues. To our knowledge, this study is the first to perform semi-automatic segmentation of the IAN using MRN images. We developed a deep learning model based on 6,027 coronal MRN images and evaluated its performance using four quantitative metrics, comparing it with six state-of-the-art models that were retrained and tested on the same dataset for small-structure segmentation. Our model achieved a dice similarity coefficient (DSC) of 0.712 ± 0.254, significantly outperforming the six comparator models. In addition, in an analysis of segmentation failure rates according to DSC thresholds, our model demonstrated the lowest failure rate. In conclusion, unlike many previous studies that focused on bony boundaries using CBCT or CT, this study demonstrates the feasibility and potential clinical utility of MRN-based IAN segmentation.

**Supplementary Information:**

The online version contains supplementary material available at 10.1038/s41598-026-45392-6.

## Introduction

The inferior alveolar nerve (IAN), a branch of the mandibular division of the trigeminal nerve, enters the mandible through the mandibular foramen and provides sensory innervation to the mandibular teeth, lower lip, and gingiva^1,2^. The IAN courses alongside the inferior alveolar artery and vein, forming the inferior alveolar neurovascular bundle within the osseous boundary known as the mandibular canal (MC)^1–3^. The IAN is frequently damaged during oral surgical procedures, including third molar extraction, dental implant surgery, and orthognathic surgery. Consequently, precise segmentation of the MC on three-dimensional imaging modalities is essential for clinicians to evaluate the spatial relationship between the MC and adjacent anatomical landmarks, thereby minimizing the risk of iatrogenic nerve injury during surgical interventions^4^.

Panoramic radiographs and cone-beam computed tomography (CBCT) are widely used in clinical practice to infer the location of the IAN by identifying the osseous boundaries of the MC^5^. However, the corticated borders of the MC are clearly visible in only approximately 60% of cases^6^, and even when visible, determination of the precise course of the IAN remains indirect. When a patient presents neurological dysfunction after implant surgery and the MC and implant fixture appear to overlap on panoramic radiography, CBCT cannot determine whether only the cortical border of the MC is disrupted, whether the internal IAN is compressed, or whether the IAN is completely transected^7^. Furthermore, such imaging does not provide information regarding the severity of nerve injury—specifically, whether the nerve itself is amputated or whether only the nerve sheath is affected—making accurate prognostic assessment of neural disturbance difficult.

Meanwhile, magnetic resonance imaging (MRI) enables high-resolution visualization of soft tissues without ionizing radiation. It has become an essential diagnostic tool not only in medicine but also in dentistry, particularly for the evaluation of temporomandibular joint discs and a variety of soft tissue lesions^5^. Although conventional MRI provides excellent soft-tissue contrast, visualization of peripheral nerves and small vessels is limited by their small diameter and signal similarity to surrounding tissues. Advances in imaging sequences have made it possible to more clearly identify small tubular structures, such as blood vessels, salivary gland ducts, and peripheral nerves. Magnetic resonance neurography (MRN) suppresses vascular signals, reduces motion and susceptibility artifacts, and enhances nerve-specific contrast by exploiting differences in T2 relaxation and proton density between neural tissue and surrounding structures^8^.

Recent studies have demonstrated the clinical utility of MRN in the evaluation of nerve pathology in both the extremities and the maxillofacial region^9–11^. In the maxillofacial region, MRN has been shown to be effective in diagnosing trigeminal neuropathy, detecting nerve edema during implant surgery, and assessing the prognosis of nerve damage following orthognathic surgery^11^. These capabilities distinguish MRN from CBCT by enabling the assessment of not only anatomical structures but also functional and pathological nerve changes.

Despite these advantages, MRN remains underutilized in the development of automated segmentation tools. Most existing artificial intelligence-based segmentation methods for the MC are derived from CBCT datasets, which inherently cannot directly visualize the nerve itself^12–22^. Direct segmentation of the IAN is of greater clinical relevance but presents substantial technical challenges due to the nerve’s small size and the difficulty of differentiating its signal intensity from that of adjacent structures^23–25^. Therefore, this study aimed to develop a deep learning model for semi-automatic segmentation of IAN on MRN images, as a feasibility investigation demonstrating the potential of MRN-based segmentation, and to evaluate its performance in comparison with six other models tested on the same dataset.

## Methods

### Patients and datasets

**Fig. 1:**
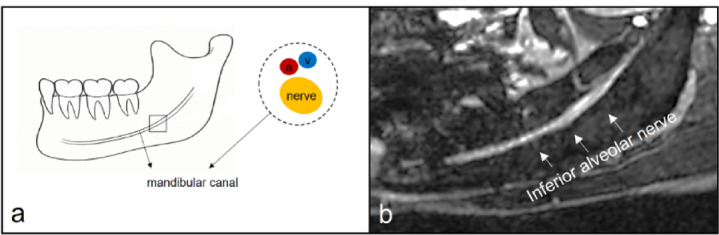
Anatomic illustration and MRN of the inferior alveolar nerve (**a**) The inferior alveolar nerve (yellow) is surrounded by the mandibular canal (black dotted line), accompanied by the inferior alveolar artery (red) and vein (blue). (**b**) The inferior alveolar nerve (white arrow) appears as a structure with high signal intensity on MRN. MRN, magnetic resonance neurography.

In this study, we used MRN images for segmentation of the IAN rather than the MC (Fig. 1). This retrospective study was approved by the Institutional Review Board of Yonsei University Dental Hospital (IRB No. 2-2024-0010). The study was conducted in accordance with the Declaration of Helsinki and relevant guidelines and regulations. The requirement for informed consent was waived by the same Institutional Review Board due to the retrospective nature of the study. All data were anonymized prior to analysis.

Among 52 patients who underwent MRN for the diagnosis of nerve disturbance at Yonsei University Dental Hospital between January 2019 and August 2024, 23 patients were excluded due to a history of oral surgery, involvement of bone pathological lesions, or poor image quality that precluded reliable identification of the IAN. Ultimately, 29 patients were included (12 males and 17 females; mean age, 48.5 ± 15.8 years; range, 13–74 years). Of the 58 IANs obtained from these 29 patients, seven IANs exhibiting pathological nerve signal were excluded. Finally, 51 IANs (6,027 images) without pathological findings were included in the analysis. Among these, 41 IANs from 26 patients with metallic dental restorations were included.

All MRN scans were acquired using a 3.0 T MRI system (Signa Pioneer, GE Healthcare, Chicago, IL, USA) with a multi-echo steady-state acquisition (MENSA) sequence. Imaging parameters included a repetition time of 20 ms, an echo time of 10 ms, and a flip angle of 30°. The field of view was 220 × 220 mm, with a matrix size of 384 × 246, resulting in a voxel size of 0.429 × 0.8 × 1.6 mm.

## IAN annotations

**Fig. 2:**
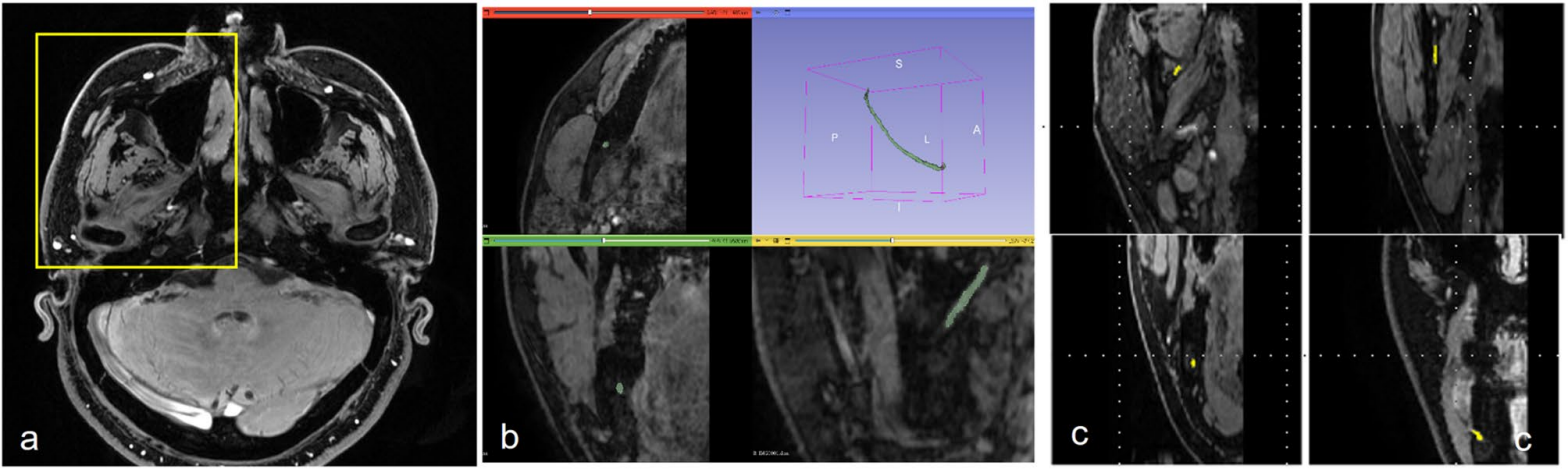
Process of annotating the inferior alveolar nerve (**a**) Axial MRN images were cropped according to predefined anatomical landmarks: the most anterior aspect of the facial buccal skin (anterior), the external acoustic meatus (posterior), the mid-palatal suture (medial), and the zygomatic arch (lateral). (**b**) On the cropped MRN image, the inferior alveolar nerve (green) is manually annotated. (**c**) The annotated mask is exported as a coronal MRN image, with the inferior alveolar nerve labeled in yellow. MRN, magnetic resonance neurography.

The IANs were manually annotated and reviewed by an oral and maxillofacial radiologist with 15 years of experience (Y.J.C.). Axial MRN images were exported in DICOM (Digital Imaging and Communications in Medicine) format, and the overall annotation workflow is illustrated in Fig. 2. Given the small size of the IAN, manual cropping of images was performed using predefined anatomical landmarks: superiorly, the cranial base; inferiorly, the most inferior point of the chin; medially, the midline palatal suture; laterally, the most lateral border of the zygomatic arch; anteriorly, the most anterior aspect of the facial buccal skin; and posteriorly, the external acoustic meatus (Fig. 2a). To ensure consistent anatomical orientation, all left-side images were horizontally flipped to match right-side orientation. Annotation of the IAN was performed from the cranial base to the mental foramen using overlapping spherical brush-based labeling to ensure anatomically continuous three-dimensional tracing in 3D Slicer (version 5.0.2; MIT, Cambridge, MA, United States). All segmentation masks were reviewed pixel by pixel in the axial, sagittal, and coronal planes (Fig. 2b). To address partial volume effects, labeling at nerve boundaries was performed conservatively. Annotated data were exported as coronal DICOM images. This approach was chosen because the coronal plane is commonly used in dentistry to assess the spatial relationship between teeth and the IAN (Fig. 2c). To assess intra-rater reliability, a second annotation was performed after a two-month interval on five IANs, representing a 10% random subset of the total dataset (51 IANs), yielding a dice similarity coefficient (DSC) of 0.7541.

## Data preprocessing

The annotated segmentation masks were converted to JPEG format through a standardized preprocessing pipeline. Pixel intensity values were extracted and normalized using min–max scaling to an 8-bit range. To preserve anatomical proportions while maintaining consistent input dimensions, zero-padding was applied. Images were subsequently resampled using the LANCZOS interpolation algorithm to fit within a 256 × 256 pixel boundary while preserving the original aspect ratio^26^. Final image dimensions of 256 × 256 pixels were obtained by centering the resized images with additional zero-padding. This preprocessing workflow yielded a uniform dataset optimized for deep learning applications.

## Model architecture

**Fig. 3:**
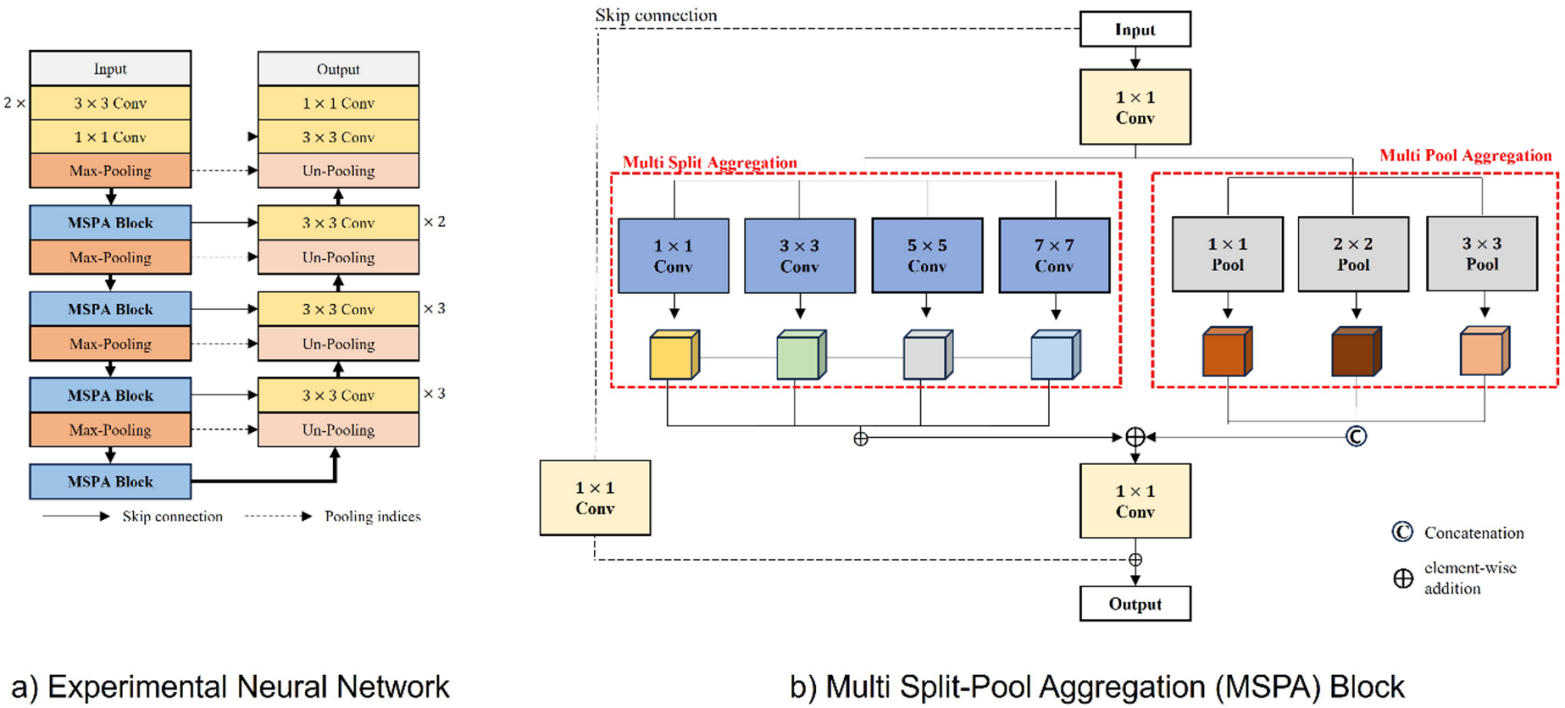
Diagram of the model used in this study.

Our model adopts a U-shaped encoder–decoder architecture that is widely used in medical image segmentation. The encoder consists of an initial convolutional stem followed by residual blocks in the style of ResNet, in which standard convolutional layers are augmented with split-attention modules introduced in ResNet^27^. Within each residual block, the split-attention operation adaptively recalibrates the importance of grouped feature channels before they are combined with identity mapping. This mechanism enhances channel-wise feature representation and improves integration within residual connections. As a result, the encoder effectively extracts both global contextual information and localized structural details (Fig. 3a). Max-pooling is employed in the encoder while retaining pooling indices, a design choice that preserves spatial accuracy and mitigates checkerboard artifacts in the decoder.

To improve segmentation of small structures, a multi split–pool aggregation (MSPA) module is incorporated at the encoder–decoder bottleneck (Fig. 3b). The MSPA module consists of two complementary components: multi split aggregation (MSA) and multi pool aggregation (MPA)^27^. The MSA component applies parallel convolutions with varying kernel sizes (1 × 1, 3 × 3, 5 × 5, and 7 × 7) to extract features across multiple spatial scales. In parallel, the MPA component captures contextual information through adaptive average pooling at multiple resolutions (1 × 1, 2 × 2, and 3 × 3)^28–30^. The resulting pooled features are refined using 1 × 1 convolutions, upsampled, and fused to strengthen local feature representation and enhance sensitivity to subtle or low-contrast regions. By integrating multi-scale feature extraction with hierarchical context aggregation, the MSPA module improves the network’s ability to segment small or low-contrast anatomical structures with greater precision and robustness. In our implementation, the MSPA module is positioned at the encoder–decoder bottleneck, where spatial resolution is lowest and high-level semantic information is concentrated, which is the stage at which small or low-contrast targets are most susceptible to information loss^31^. Compared with conventional spatial pyramid modules, MSPA jointly models multi-kernel and multi-resolution features, thereby better preserving thin boundaries.

The decoder progressively reconstructs the segmentation map through max-unpooling operations guided by pooling indices generated in the encoder. Skip connections between corresponding encoder and decoder layers facilitate retention of spatial details lost during downsampling^32^. Each decoding stage is followed by batch normalization and ReLU activation to stabilize training and enhance representational capacity^33^. This architectural design enables effective integration of semantic abstraction and spatial detail, which is particularly advantageous for segmenting small and complex anatomical structures such as the IAN. By combining multi-scale receptive fields with localized context aggregation, the proposed model improves boundary delineation and increases sensitivity to subtle morphological variations. Consequently, the model is well suited for medical image segmentation tasks involving fine anatomical details and spatially constrained structures that are often challenging to detect using conventional approaches^29^.

## Training process

Random sampling was employed to preserve the distribution across the dataset. Owing to the relatively small dataset size, a 10-time repeated random split validation strategy (Monte Carlo cross-validation) was adopted instead of conventional k-fold cross-validation. All data splits were performed at the patient level (70% training, 15% validation, and 15% testing) to prevent data leakage between the left and right IANs from the same patient.

For model performance comparison, our model and six state-of-the-art networks (CaraNet^34^, DS-TransUNet^35^, DUCKNet^36^, DeepLabV3 +^37^, HarDNet-MSEG^38^, and MEGANet^39^ were implemented using the PyTorch library (version 1.10.1), and all experiments were conducted on an NVIDIA GeForce RTX 3090 GPU with 24 GB of memory. Optimization was performed using the AdamW optimizer with an initial learning rate of 1 × 10⁻³ and a weight decay of 1 × 10⁻⁴, together with a cosine annealing learning-rate scheduler. Each model was trained for 50 epochs with a batch size of 32. The Focal Tversky Loss (γ = 2.0; mean-reduction mode) was employed to address the severe class imbalance between nerve and background pixels. Early stopping with a patience of 25 epochs was applied to mitigate overfitting. The inference time of our model was approximately 0.018 s per image slice, and the total training time was 0.59 h. The inference time and total training time of each model are summarized in Table S1.

### Model evaluation

Segmentation performance was evaluated using four metrics^40^: DSC, intersection over union (IoU), precision, and recall. Their definitions are as follows:

$$\:\mathrm{D}\mathrm{S}\mathrm{C}\:=\:\frac{2\:*\:TP}{TP\:+\:FP\:+\:FN}$$

$$\:\mathrm{I}\mathrm{o}\mathrm{U}\:=\:\:\frac{TP}{TP\:+\:FP\:+\:FN}$$

$$\:\mathrm{P}\mathrm{r}\mathrm{e}\mathrm{c}\mathrm{i}\mathrm{s}\mathrm{i}\mathrm{o}\mathrm{n}\:=\:\frac{TP}{TP\:+\:FP}$$

$$\:\mathrm{R}\mathrm{e}\mathrm{c}\mathrm{a}\mathrm{l}\mathrm{l}\:=\:\frac{TP}{TP\:+\:FN}$$

where TP: true positive, TN: true negative, FP: false positive, and FN: false negative.

Given the limitations associated with evaluating small-structure segmentation using only four quantitative metrics, the segmentation failure rate was additionally analyzed. Because the IAN is a very small tubular structure that extends continuously across multiple MRN slices, segmentation failures were categorized into three subtypes based on DSC values: complete failure, tracking failure, and boundary delineation failure. Complete failure was defined as absence of any predicted mask (DSC = 0), tracking failure as discontinuous or anatomically deviated predictions (DSC < 0.2), and boundary delineation failure as over- or under-segmentation resulting in loss of clinical relevance (DSC < 0.3). The proportion of each failure subtype was calculated for all models. Statistical significance was assessed using the Wilcoxon signed-rank test with Bonferroni correction. All analyses were conducted in Python (version 3.9; Python Software Foundation, https://www.python.org/).

## Results

**Fig. 4:**
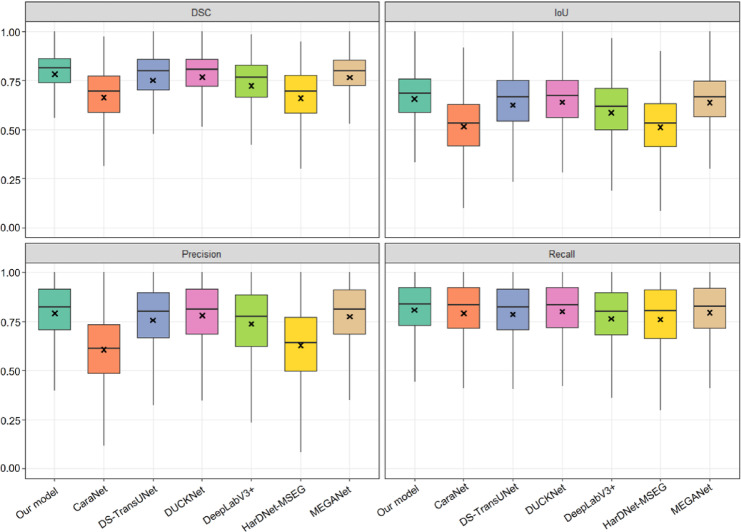
Box plot of segmentation performance of our model compared to other models DSC, dice similarity coefficient; IoU, intersection over union Mean values are indicated by the symbol “×” within each box plot. Our model demonstrated the best performance compared with the other models, with statistically significant differences across all metrics (Wilcoxon signed-rank test with Bonferroni correction, *p* < 0.05).

In our model, the mean DSC, IoU, precision, and recall values were 0.712 ± 0.254, 0.598 ± 0.235, 0.722 ± 0.275, and 0.736 ± 0.275, respectively. Our model demonstrated significantly higher performance than all other models across all four evaluation metrics (Wilcoxon signed-rank test with Bonferroni correction, *p* < 0.05). MEGANet^39^ exhibited the second-best overall performance, whereas HarDNet-MSEG^38^ yielded the lowest DSC, IoU, and recall values. Precision was lowest in CaraNet^34^. The segmentation performance of each model is summarized in Table 1; Fig. 4, with detailed statistical comparisons provided in Supplementary Table S2.

**Table 1:** Performance comparison compared with six other models using four metrics.

Model	DSC Mean ± SD (median)	IoU Mean ± SD (median)	Precision Mean ± SD (median)	Recall Mean ± SD (median)
**Our model**	**0.712 ± 0.254 (0.8000)**	**0.598 ± 0.235(0.6667)**	**0.722 ± 0.275 (0.8056)**	**0.736 ± 0.275 (0.8193)**
CaraNet^34^	0.597 ± 0.248 (0.6744)	0.463 ± 0.217 (0.5088)	0.545 ± 0.251(0.5849)	0.712 ± 0.296 (0.8108)
DS-TransUNet^35^	0.674 ± 0.274 (0.7833)	0.559 ± 0.252 (0.6438)	0.679 ± 0.293(0.7750)	0.704 ± 0.295 (0.8030)
DUCKNet^36^	0.678 ± 0.278 (0.7869)	0.565 ± 0.253 (0.6486)	0.689 ± 0.299 (0.7857)	0.708 ± 0.302 (0.8088)
DeepLabV3 + ^37^	0.647 ± 0.266 (0.7467)	0.525 ± 0.240 (0.5957)	0.660 ± 0.291 (0.7419)	0.685 ± 0.293 (0.7759)
HarDNet-MSEG^38^	0.594 ± 0.251 (0.6750)	0.461 ± 0.219 (0.5094)	0.566 ± 0.265 (0.6122)	0.684 ± 0.299 (0.7778)
*MEGANet* ^39^	0.694 ± 0.261 (0.7912)	0.579 ± 0.240 (0.6545)	0.703 ± 0.285 (0.7895)	0.722 ± 0.280 (0.8065)

Our model showed the best performance, with a significant difference in all metrics (Wilcoxon signed-rank test with Bonferroni correction, *p* < 0.05). The second best model was MEGANet^39^. Detailed *p* values are provided in Supplementary Table S2.

DSC, dice similarity coefficient; IoU, intersection over union; SD, standard deviation.

Our model demonstrated the lowest failure rates across all failure subtypes, with complete failure at 8.9%, tracking failure at 9.5%, and boundary delineation failure at 9.9% (Table 2). MEGANet^39^ showed the second-lowest overall failure rate, whereas DUCKNet^36^ exhibited the highest rates of both complete and tracking failures (Table 2). In contrast to our model, which demonstrated stable failure rates across subtypes, the other models showed a pronounced increase in the proportion of boundary delineation failures compared with tracking failures.

**Table 2:** The segmentation failure rates of all seven models.

Model	Complete failure (%)	Tracking failure (%)	Boundary delineation failure (%)
Our model	**8.9**	**9.5**	**9.9**
CaraNet^34^	10.1	11.5	13.3
DS-TransUNet^35^	10.4	11.8	12.9
DUCKNet^36^	11.7	12.3	12.9
DeepLabV3 + ^37^	10.5	11.8	12.8
HarDNet-MSEG^38^	10.1	12.0	13.7
*MEGANet* ^39^	9.3	10.3	11.0

Complete failure was defined as DSC = 0, and Tracking failure was defined as DSC < 0.2, Boundary delineation was defined as DSC < 0.3.

Our model showed the lowest failure rate, followed by MEGANet^39^.

DSC, dice similarity coefficient.

**Fig. 5:**
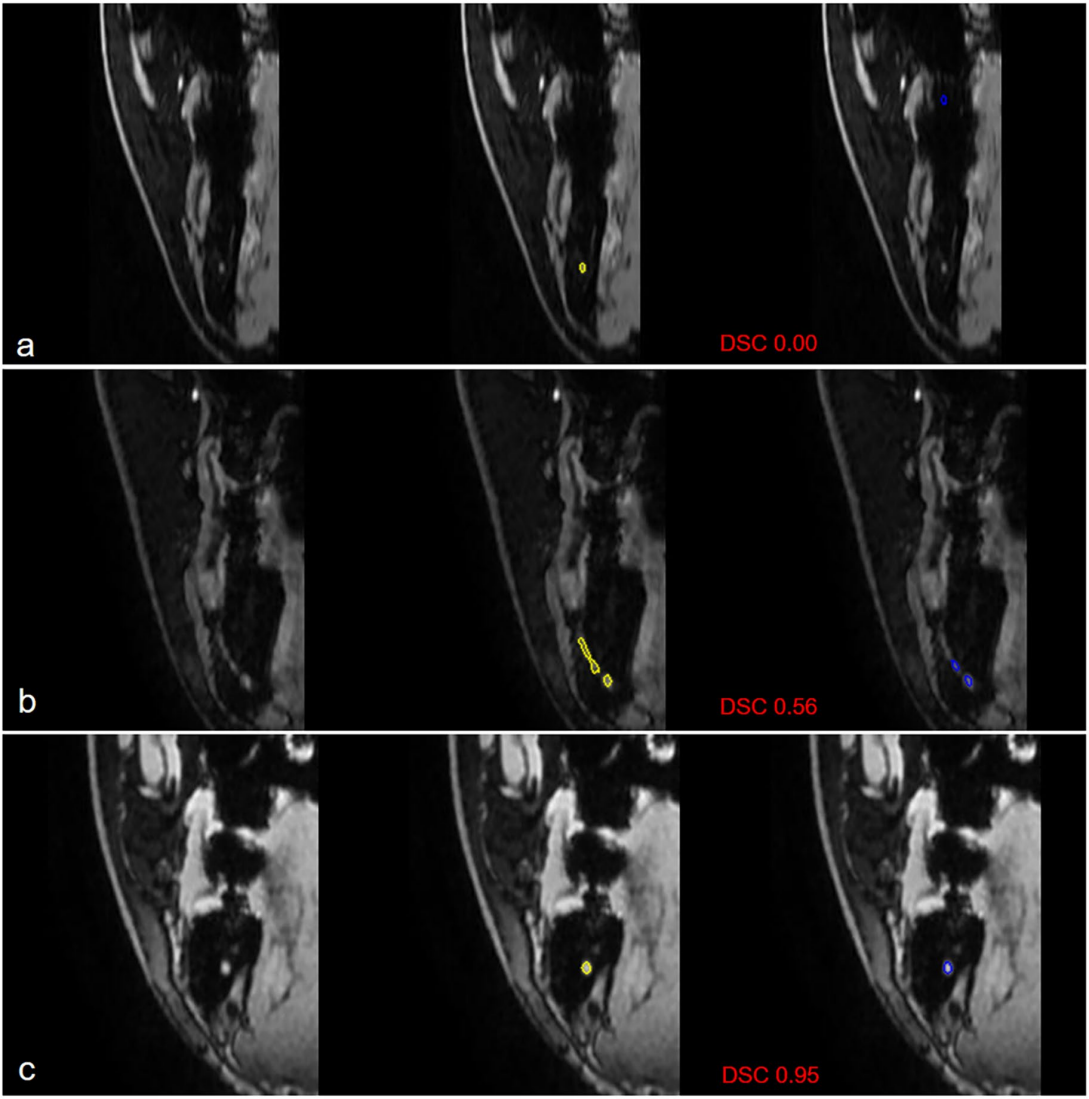
Visualization of segmentation results across different DSCs Input images, ground truth (yellow), and predicted segmentations (blue) are shown for cases with different DSC values, representing complete failure (**a**) below-average segmentation performance (**b**) and above-average accurate segmentation (**c**) respectively. DSC, dice similarity coefficient.

**Fig. 6:**
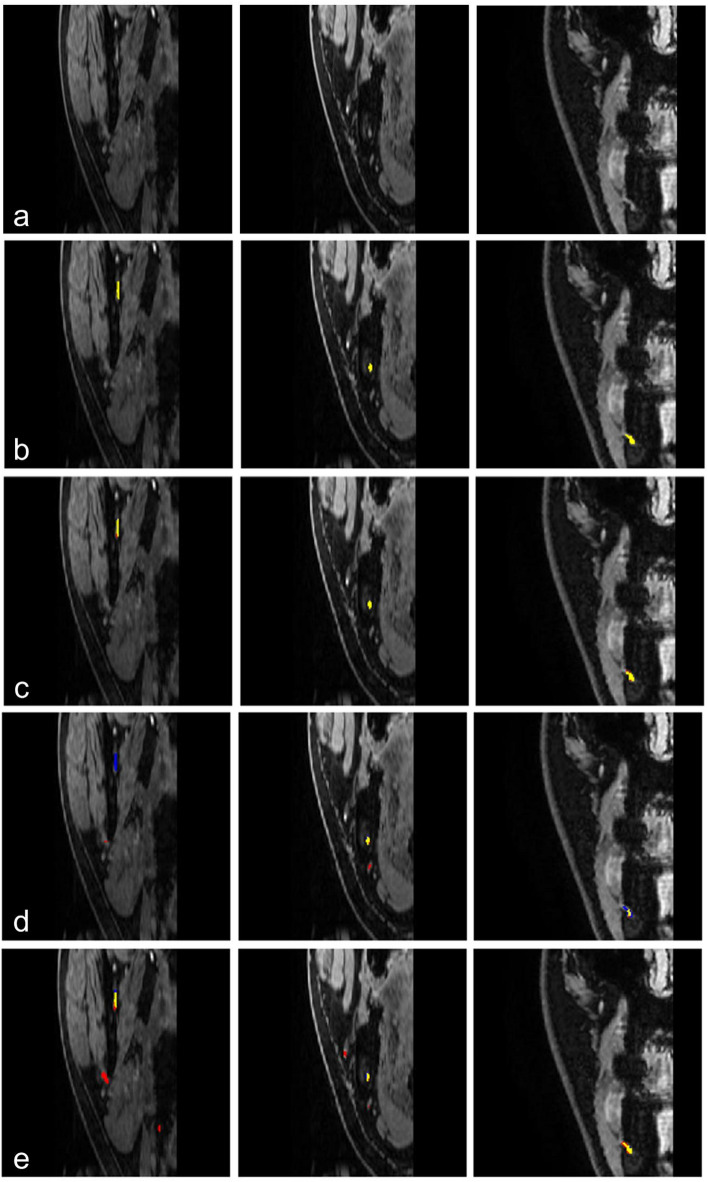
Visualization of segmentation results on representative image sections Each column presents the same representative image sections to qualitatively illustrate model performance. Predicted regions are shown in red, ground truth in blue, and overlapping regions in yellow. (**a**) Input images. (**b**) Ground truth. (**c**) Our model. (**d**) DeepLabV3 +^37^. (**e**) MEGANet^39^ Visualization of all seven models are provided in Supplementary Fig. S1.

In one IAN, all models exhibited complete failure across the entire image series. Representative segmentation results of our model across varying DSC values, including a complete failure case, are illustrated in Fig. 5. The ground truth and segmentation results of the seven models were visualized for comparison on representative image sections (Fig. 6 and Supplementary Fig. S1).

## Discussion

In this study, we developed an experimental neural network for semi-automatic segmentation of the IAN on MRN images. Using MRN, Balsiger et al. performed semi-automatic segmentation of the sciatic nerve and reported DSC values of 0.859 in healthy volunteers and 0.719 in patients, which were comparable to the level of human inter-rater agreement^24^. Similarly, our model achieved a DSC of 0.712 ± 0.254, an IoU of 0.598 ± 0.235, precision of 0.722 ± 0.275, and recall of 0.736 ± 0.275. Given the inherent variability in human manual annotation of small anatomical structures, these results suggest that our model performs near the upper limit achievable by human experts.

This study has two main contributions. First, this study is the first to automatically segment the IAN on MRN, directly visualizing the nerve rather than the MC. Assessment of the IAN within the mandible has traditionally depended on identification of the MC; therefore, most previous studies have focused on automatic segmentation of the MC using CT or CBCT^12–22^. Although CBCT offers advantages such as small voxel size, low radiation dose, and relatively low cost, the MC is often indistinctly visualized in patients with systemic diseases associated with reduced bone density. In contrast, MRN directly visualizes peripheral nerves by accentuating the prolonged T2 signal of endoneurial fluid and nerve fascicles, which appear hyperintense on T2-weighted or other nerve-specific sequences^8^. Moreover, MRN provides quantitative signal changes related to the chemical composition of nerve tissue, thereby reflecting its pathological condition^9–11^.

Previous studies on automatic segmentation of MC using CT or CBCT have demonstrated a wide range of performance. In heterogeneous datasets with poor canal visibility, reported DSC values range from 0.55 to 0.75, whereas under more controlled conditions, DSC values as high as 0.80–0.90 have been reported, with the best performance reaching 0.903 using CBCT datasets^12–22^. However, MRI has disadvantages for segmenting small anatomical structures. Improving spatial resolution requires smaller voxel sizes, which inevitably reduce the signal-to-noise ratio and limit the effective contrast available for delineating fine anatomical structures^23,41^., including relatively low spatial resolution, partial volume effects, and reduced signal-to-noise ratio when smaller voxel sizes are used^23^. Additional challenges arise from motion artifacts and low intrinsic contrast between nerves and surrounding soft tissues. Consequently, although reducing voxel size in MRN improves nerve delineation, it inherently compromises overall image quality and diagnostic effectiveness for adjacent structures. Despite these inherent limitations of MRN, our model achieved a DSC of 0.712 ± 0.254, comparable to results reported in previous studies of MC segmentation using CT or CBCT.

Second, our model performed statistically significantly better than six state-of-the-art models across four evaluation metrics. The DSC, which represents the harmonic mean of precision and recall, indicates that our model effectively minimized background noise while accurately identifying IAN pixels. In addition, MEGANet^39^ demonstrated strong performance, particularly with high recall values, suggesting robust continuity of nerve detection. In contrast, HarDNet-MSEG^38^ and CaraNet^34^ exhibited the lowest performance, frequently over-segmenting adjacent tissues due to low precision. Compared with these models, our approach achieved a more balanced trade-off between precision and recall, thereby minimizing both under- and over-segmentation. This balance represents a key aspect of the proposed model’s clinical applicability.

Based on segmentation failure analysis categorized into three subtypes according to DSC thresholds, our model achieved the lowest failure rates: 8.9% for complete failure (DSC = 0), 9.5% for tracking failure (DSC < 0.2), and 9.9% for boundary delineation failure (DSC < 0.3). DUCKNet^36^, which showed intermediate overall performance, exhibited the highest complete (11.7%) and tracking failure rates (12.3%). Unlike HarDNet-MSEG^38^ and CaraNet^34^, which demonstrated a pronounced increase in boundary delineation failure compared with tracking failure, our model maintained relatively stable failure rates across all subtypes. It suggested that boundary delineation errors were the primary source of segmentation failure.

In our dataset, of the total 51 IANs, 41 IANs had several image sections affected by artifact-induced dental restorations. In one IAN, all models, including our model, showed segmentation failure across the entire image series. This failure was attributed to the combined effects of metal artifacts from the implant crown–fixture complex and motion artifacts. However, even in the presence of metal artifacts, several cases demonstrated DSC values of 0.9 or higher, in which the artifacts were primarily localized around the crown of the tooth and did not directly affect the MC. These findings suggest that our model exhibits clinically applicable performance in cases of dental restorations that do not invade or compromise the IAN.

Although this study demonstrated the feasibility of MRN-based IAN segmentation, several limitations should be acknowledged within the scope of this work. First, due to the high cost and technical complexity of MRN, the dataset was limited in size and derived from a single institution. Given the small size of the IAN relative to the overall image and the low signal-to-noise ratio of MRN images, the region of interest was manually cropped prior to segmentation. Therefore, the proposed model should be considered a semi-automatic segmentation framework. In addition, three-dimensional or 2.5-dimensional modeling approaches could not be implemented. In future studies, model performance may be enhanced using larger, multi-institutional datasets, centerline-tracking–based post-processing strategies, and further advancement toward fully automatic three-dimensional model development. Second, pathological IANs and the lingual nerve were not included because of their potential confounding effects and poor visibility. To minimize the impact of signal alterations or discontinuities associated with pathological nerve changes on segmentation performance, only normal nerves were included in this study. Third, without region-specific analysis, the model’s performance was evaluated across the entire image set. Considering anatomical variations, such as the anterior loop around the mental foramen, performance analysis according to the anatomical location of the IAN should be considered. To further improve model performance, future studies should incorporate region-specific augmentation or sampling strategies, as well as apply fine-tuning methods. Although motion and susceptibility artifacts remain challenging, future work including motion correction, accelerated acquisition strategies, and advanced metal artifact reduction techniques using deep learning–based reconstruction may further improve segmentation stability under artifact-prone imaging conditions^42,43^.

In conclusion, our model shows strong promise for semi-automatic segmentation of the IAN on MRN, a modality that enables nerve-specific signal assessment. While CBCT and CT have traditionally been used to assess the anatomical location of the IAN within the mandible, MRN provides complementary information by characterizing signal changes within the nerve itself. To our knowledge, this is the first study to apply deep learning for semi-automatic segmentation of the IAN using clinically acquired MRN in dentistry. This approach may reduce the workload associated with manual annotation, assist clinicians in surgical planning, and represent a meaningful step toward integrating deep learning into dental imaging workflows.

## Supplementary Information

Below is the link to the electronic supplementary material.

Supplementary Material 1

Supplementary Material 2

Supplementary Material 3

## Data Availability

The code for model training and evaluation is publicly available at https://github.com/HaeSung-Oh/IAN-MRN-Segmentation. Datasets in the study are available from the corresponding author upon reasonable request, subject to approval by the institutional review board.
