# Supplementary material for: A feasibility study of deep learning-based segmentation of the inferior alveolar nerve on magnetic resonance neurography
Source: Sci Rep. 2026 Apr 1;16:15433. doi: 10.1038/s41598-026-45392-6 (PMC13184129; doi:10.1038/s41598-026-45392-6)
Supplement: Supplementary file 1 — Supplementary Material 1 [file 41598_2026_45392_MOESM1_ESM.docx]

**Table S2. Bonferroni-corrected p-values for model results comparisons**

| Models | DSC | IoU | Precision | Recall |
| --- | --- | --- | --- | --- |
| CaraNet^34^ | <0.001 | <0.001 | <0.001 | <0.001 |
| DS-TransUNet^35^ | <0.001 | <0.001 | <0.001 | <0.001 |
| DUCKNet^36^ | <0.001 | <0.001 | <0.001 | <0.001 |
| DeepLabV3+^37^ | <0.001 | <0.001 | <0.001 | <0.001 |
| DeepLabV3+^37^ | <0.001 | <0.001 | <0.001 | <0.001 |
| HarDNet-MSEG^38^ | <0.001 | <0.001 | <0.001 | <0.001 |
| MEGANet^39^ | <0.001 | <0.001 | <0.001 | <0.001 |

DSC, dice similarity coefficient; IoU, intersection over union; SD, standard deviation

P-values were calculated using the Wilcoxon signed-rank test with Bonferroni correction. All comparisons showed statistically significant differences (p < 0.05).
