# Supplementary material for: A feasibility study of deep learning-based segmentation of the inferior alveolar nerve on magnetic resonance neurography
Source: Sci Rep. 2026 Apr 1;16:15433. doi: 10.1038/s41598-026-45392-6 (PMC13184129; doi:10.1038/s41598-026-45392-6)
Supplement: Supplementary file 2 — Supplementary Material 2 [file 41598_2026_45392_MOESM2_ESM.docx]

| Model | Inference Time (ms/slice) | Training time (hour) |
| --- | --- | --- |
| Our model | 18.2 ± 1.3 | 0.59 |
| CaraNet^34^ | 61.0 ± 19.9 | 0.82 |
| DS-TransUNet^35^ | 54.2 ± 21.0 | 2.18 |
| DUCKNet^36^ | 103.1 ± 22.7 | 3.34 |
| DeepLabV3+^37^ | 24.2 ± 1.3 | 0.50 |
| HarDNet-MSEG^38^ | 33.3 ± 21.2 | 0.67 |
| MEGANet^39^ | 37.2 ± 1.5 | 0.69 |

**Table S1**. Inference time and training time on each model
